# Proteomic characterization of adult *Fasciola gigantica* recovered from naturally infected buffaloes in Punjab Pakistan

**DOI:** 10.1371/journal.pone.0356497

**Published:** 2026-08-21

**Authors:** Kinza Shahid, Mirza Imran Shahzad, Muhammad Ehsan, Rui-Si Hu, Areeba Yousaf, Gildardo Rivera, Muhammad Safdar, Muhammad Rashid, Muhammad Irfan Malik, Muhammad Mudasser Nazir

**Affiliations:** 1 Department of Parasitology, Faculty of Veterinary and Animal Sciences, The Islamia University of Bahawalpur, Punjab, Pakistan; 2 Department of Biochemistry and Molecular Biology, Institute of Biochemistry, Biotechnology and Bioinformatics, The Islamia University of Bahawalpur, Punjab, Pakistan; 3 School of Health and Wellness Industry & School of Medicine, Sichuan University of Arts and Science, Dazhou, Sichuan, China; 4 Laboratorio de Biotecnología Farmacéutica, Centro de Biotecnología Genómica, Instituto Politécnico Nacional, Reynosa, Tamaulipas, México; Mekdela Amba University, ETHIOPIA

## Abstract

**Background:**

*Fasciola gigantica (F. gigantica*) is a widespread zoonotic trematode that poses a substantial burden on livestock and public health in endemic regions. Elucidating host–parasite interactions relies largely on proteomic data. Excretory-Secretory proteins (ESPs) can play central roles in the host-parasite interactions, immune evasion, and parasite pathogenicity; however, proteomic information on *F. gigantica* infecting buffalo remains scarce, particularly from Pakistan.

**Objectives:**

In this study, we present the first comprehensive proteomic characterization of adult *F. gigantica* recovered from naturally infected buffaloes in Punjab, Pakistan, with a specific focus on putative excretory–secretory proteins.

**Methodology:**

Somatic protein extracts from adult flukes were initially assessed using sodium dodecyl sulfate–polyacrylamide gel electrophoresis (SDS–PAGE) and Western blotting, confirming a high abundance and broad molecular weight distribution of parasite proteins. Subsequent liquid chromatography–tandem mass spectrometry (LC–MS/MS) analysis generated the largest proteomic dataset for *F. gigantica* to date, a total of 4,777 proteins. *In‒silico* prediction using DeepLoc 2.0, SignalP, and transmembrane helices (TMHMM), enabled the identification of a substantial repertoire of putative ESPs, including both classical secretory proteins possessing N-terminal signal peptides and non-classical secreted proteins lacking transmembrane helices. Functional annotation and Gene Ontology (GO) enrichment analysis revealed that these proteins were predominantly associated with catalytic, hydrolase, and oxidoreductase activities, highlighting their roles in tissue invasion and nutrient acquisition.

**Conclusions:**

Among 4,777 proteins, 95 proteins associated with molecular functions, 77 in biological processes, and 65 in cellular components were linked to immune regulation, stress response, metabolic adaptation, and parasite survival. Pathway enrichment further highlighted their involvement in protein processing, signal transduction, and mechanisms facilitating host invasion and chronic infection. Collectively, these findings emphasize the vital role of proteins (somatic/putative secreted) in orchestrating host–parasite interactions and sustaining chronic infection.

## 1. Introduction

Fascioliasis has been considered as one of the well-known neglected tropical diseases (NTDs) caused by *Fasciola gigantica* and *Fasciola hepatica*, which infects sheep, goats, cattle, and other herbivorous animals including humans [1], leading to significant financial and health consequences accumulating 3-billion US$ annually [2]. To distinguish between *F. gigantica* and *F. hepatica* the two species’ non-overlapping size ranges were evident from the comparison of ventral sucker-posterior (VS-P) measurements, with *F. hepatica* (8.86–25.08 mm) continuously displaying lower values than *F. gigantica* (26.28–50.09 mm). The reliability of VS-P as a diagnostic morphometric indicator for species differentiation was supported by this clear separation as well as their innate body size variability [3]. According to estimates by the World Health Organization (WHO), fascioliasis constitutes a significant global public health concern, with more than 2.4 million individuals currently infected across over 70 countries spanning Asia, Africa, the Americas, and Europe [4]. Furthermore, the disease placed an estimated 180 million people at risk of infection worldwide, with the total number of affected individuals projected to exceed 17 million, underscoring its substantial epidemiological burden [5].

Research focusing on the region establishes that *F. gigantica* is overwhelmingly the primary etiological agent of fascioliasis in Punjab, circulating in cattle, buffalo, sheep, and goats. Studies utilizing multi-marker analyses—including the Internal Transcribed Spacer (ITS-1 and ITS-2) of ribosomal DNA, mitochondrial nad1 and cox1 markers, and nuclear markers (*pepck* and *pold*)—have identified high levels of genetic substructure and novel haplotype sequences in Pakistani *F. gigantica* populations. Mitochondrial genetic analyses suggest distinct genetic differences between *F. gigantica* specimens isolated from buffaloes compared to those from goats [6]. Phylogenetic and network analyses indicate that the *F. gigantica* strains in Pakistan cluster closely with Indian and Chinese clades, pointing toward distinct historical origins and dispersal patterns of the parasite in the region [7].

Fascioliasis is characterized by a complex heteroxenous life cycle that involves aquatic snails of the family *Lymnaeidae* as intermediate hosts, within which the parasite undergoes successive asexual developmental stages, including miracidia, sporocysts, rediae, and cercariae [8]. The released cercariae subsequently encyst as metacercariae on aquatic vegetation and are ingested by the definitive vertebrate host, where the juvenile flukes excyst, migrate to the hepatobiliary system, and mature into adult worms that reproduce sexually [9]. To date, no commercially available vaccine has demonstrated consistent efficacy in preventing fascioliasis. Moreover, the increasing emergence of anthelmintic resistance, particularly to triclabendazole, has become a major impediment to effective therapeutic control of the disease [10].

*Fasciola* species predispose both humans and animal hosts to co-infection with other pathogens, a phenomenon largely attributed to parasite-induced immunomodulation [5]. Although anthelmintic treatment can reverse immunoregulatory following parasite clearance, the persistence of immune suppression during infection indicates that *Fasciola* actively maintains an immunosuppressive state. To aid in its survival and the development of a persistent infection, the liver fluke *F. gigantica* modifies a number of signaling pathways in infected buffaloes. Buffaloes trigger both innate and adaptive immune responses in reaction to the parasite invasion in order to combat the infection. Thus, novel proteins that may be involved in the host–parasite dynamics but may also serve as potential biomarkers for the early detection, diagnosis, and monitoring of fascioliasis [11]. In this context, parasite-derived excretory–secretory products released during host-parasite interplay profoundly interfere with multiple layers of host immunity, ranging from early pathogen recognition to terminal effector responses, thereby enabling helminths to function as exceptionally potent immunoregulators [12].

The complex adaptations required by the parasitic way of life are represented by the range of proteins released or secreted by parasites [13]. A variety of protein compositions of excretory–secretory proteins (ESPs) has been investigated from of species, including *F. hepatica* [14], *F. gigantica* [15], *Ancylostoma caninum* [16]*, Heligmosomoides polygyrus* [17], *Dirofilaria immitis* [18]*, Ascaris suum* [19], *Haemonchus contortus* [20], and *Nippostrongylus brasiliensis* [21], through mass spectrometry analysis. Dynamic stage-specific variations in ESPs across different phases of infection can be systematically interrogated using integrated proteomic and bioinformatics approach.

Such analyses enable the high-throughput identification of key antigenic molecules potentially involved in immune evasion, immunomodulatory mechanisms, and the induction of host immune responses. Proteomic analysis of *Toxocara canis* revealed that the larval extract contained 64 ESPs and 582 somatic proteins [22]. Subsequent sequencing of the *T. canis* genome enabled *in silico* prediction of the secretome, estimating a total of 870 ESPs [23]. In *H. polygyrus*, 209 proteins were classified as ESPs, including globins, vitellogenin homologs, fatty acid and retinol-binding proteins, and proteins associated with the allergen V5/Tpx-1 family. These findings further suggest the presence of multiple immunomodulatory molecules within the parasite secretome [17].

Recent proteomic and extracellular vesicle (EV) investigations have improved understanding of *F. hepatica* biology, showing the extensive protein repertoire involved in host-parasite interactions. EVs secreted from the tegument and gut convey a variety of chemicals involved in immune regulation and parasite persistence [24]. Furthermore, extracellular vesicle (EV) studies have indicated that *F. hepatica* produces separate EV populations from the gastrodermis and tegument, containing proteins related with parasite biology, including cathepsin L zymogens [25]. These findings indicate EV-mediated communication and protease-driven metabolism as critical processes for parasite adaptability and pathogenicity.

Pre-genomic methods have discovered vaccine candidates with different protection rates. Sub-proteomics provides new opportunities to systematically uncover the relative contribution of specific family proteins to vaccine formulations among populations, since many candidates belong to protein superfamilies [26]. Recent proteomic investigations have highlighted the complexity of helminth somatic and secretory proteomes. For instance, a study on crude somatic extracts of adult *Toxocara cati* identified 363 proteins [27], while the majority of the 34 proteins examined in a subsequent analysis were already confirmed to be conserved across helminth parasites [28]. Similarly, the ES proteome of *A. caninum* was reported to comprise 105 proteins [16]. Proteases represented a dominant functional group within nematode secretomes, with cysteine, aspartic, and metalloproteases constituting the three principal classes [29]. To systematically identify and annotate predicted ES proteins encoded by the *Meloidogyne incognita* genome, multiple open-source bioinformatics tools were employed, resulting in the prediction of 1,889 ES proteins, the most comprehensive *in silico* secretome dataset reported for this species to date. The Gene Ontology (GO) annotation analysis of the functional information of the predicted 1889 ES proteins revealed an overrepresentation of binding (GO:0005488), catalytic (GO:0003824), and hydrolase activities (GO:0016787), which are hallmark features of secreted proteins and are closely associated with host–parasite interactions [30].

A detailed protein profile of *F. hepatica* highlighted total of 776 proteins at outer and internal layers of the parasite that were shown to be drug targets for other parasites [31]. Huang et al., (2019) designed a proteomic study at different infection stages, and identified 147 at 42 days post infection, 119 at 70 dpi, and 130 at 98 dpi,which include cathepsin family, CaBPs, and GSTs [32]. In a similar proteomic study, the differentially abundant proteins [18] were confirmed via PRM analysis of first simultaneous protein profile of different organs of buffaloes infected with *F. gigantica* [33]. Numerous regulatory proteins within the parasite’s proteome have the capacity to modulate host immunity by altering or suppressing immune responses, through the development of receptor-ligand complexes on host cell surface [34]. Studied have further demonstrated that Fg-ESPs play a pivotal role in immune modulation during the parasite-host relationship [35,36].

Proteomic investigation provides a comprehensive characterization of the parasite’s protein repertoire, offering valuable insights for the development of more effective interventions against fasciolosis. [31]. Notably, the proteases in proteins secreted by infectious larvae, juvenile, and adult flukes corresponds closely with the parasite’s progression through host tissues and its interactions with diverse host macromolecules [37]. The identification and validation of novel targets for therapeutics or vaccines have become increasingly urgent due to the emergence of anthelmintic resistance. To uncover promising the molecular targets for anti-parasitic interventions, extensive studies have focused on proteases of various trematodes, particularly schistosomes and liver flukes, with ongoing research continuing to expand our understanding of their functional roles [38]. The objective of this study was to characterize the comprehensive proteomic profile of *F. gigantica* based on peptide level evidence, with a particular focus on predicting ESPs. These proteins represent promising targets for future vaccine development.

## 2. Materials and methods

**Ethical approval statement:** Samples were collected from a local slaughterhouse following routine inspection procedures, and no live animal was involved. All the experiments were conducted under the project, in compliance with the Animal ethics committee, Office of Research Innovation and Commercialization (ORIC) (Approval no. 461/ORIC) and BioSafety committee (IBSC) (Approval no. 473/ORIC), of The Islamia University of Bahawalpur.

### 2.1. Parasite collection

The liver samples were collected from naturally infected buffaloes slaughtered at different abattoirs in Punjab, Pakistan. The buffaloes were of Nili Ravi breed commonly found in Punjab. The animals grazed mostly within municipal pastures and areas near slaughterhouses. Adult *F. gigantica* specimens were collected at three independent sampling time points from naturally infected buffaloes. At each time point, approximately 30–35 slaughtered buffaloes were examined, and 200–400 adult flukes were recovered from a single infected host. Parasites collected at each sampling event constituted an independent biological pool, yielding three biological replicates for downstream proteomic analyses. All recovered flukes were identified as *F. gigantica* based on detailed morphological examination. Consequently, molecular confirmation was not repeated in the present study because the species identity had already been established, [6] and the objective of this work was to evaluate the proteomic profiles of morphologically confirmed *F. gigantica* specimens, clearly distinguishing *F. gigantica*, which exhibits an elongated body morphology, from *F. hepatica*, which is comparatively shorter and broader in shape. Flukes were collected post-mortem after slaughter. Prior to protein extraction, freshly recovered parasites were properly washed several times with sterile phosphate-buffered saline (PBS) to remove residual host materials such as blood, bile, and liver tissue debris. Three flukes, one from each of three different buffaloes, were selected for subsequent protein isolation and analysis. After careful inspection for adult parasites in the gall bladder and hepatic veins, the flukes were retrieved, and thoroughly washed in phosphate buffer saline (PBS, pH 7.4) to remove host tissue debris and blood contaminants. Adult flukes were homogenized in 500 μL lysis buffer containing 8 M urea, 50 mM Tris-HCl (pH 8.0), 1% protease inhibitor cocktail, and 1 mM dithiothreitol (DTT) to ensure complete protein solubilization. Homogenates were sonicated on ice and centrifuged at 14,000 ×g for 30 min at 4°C to remove insoluble debris. Protein concentrations were determined using a Bicinchoninic Acid (BCA) assay kit (2.75-3.35µg/µL) (Thermo Scientific™, Waltham, MA, USA). Supernatant was transferred to another tube and then it was freeze-dried into powder and stored at ‒80 ^o^C.

### 2.2. Proteins identification through sodium dodecyl sulfate–polyacrylamide gel electrophoresis (SDS-PAGE) and western blotting

According to the results of protein concentration determined, an equal amount of protein (2.75x20=55 μg) was taken from each sample into centrifuge tubes, 5 μL 4 × loading buffer, and 2% SDS were added to make the final volume 25 μL (20 μL sample and 5 μL loading buffer). Following electrophoresis, the gel was stained with Coomassie Brilliant Blue for one hour and subsequently de-stained for six hours, with the destaining solution changed every two hours. After completion of staining, the protein band pattern was visualized using a Dual LED Blue/White Light Transilluminator (Model: LB0100) to assess protein separation.

Western blotting was subsequently performed to assess the immunogenicity of *Fg*ESPs. The separated proteins were transferred onto a nitrocellulose membrane, which was then blocked with 5% skim milk (0.75g skim milk in 15mL TBST) (Sigma-Aldrich, Hamburg, Germany) for 1 hour in 4°C to prevent nonspecific binding. After blocking, the membrane was washed three times (5 min/time) and incubated with the primary antibody (20 μL primary antibody in 10mL TBST or in skim milk) overnight at 4°, which was obtained from *Fasciola* positive buffalo. A negative control lane (C) containing no sample or primary antibody was included. Following primary antibody incubation, the membrane was washed three times (5 min/time) and incubated with horseradish peroxidase (HRP)–conjugated rabbit anti-bovine IgG polyclonal secondary antibody (1:20000, Invitrogen, USA) (10 μL secondary antibody in 10mL TBST or 5% BSA in TBST) for 2 hrs with shaking at 80 rpm. After incubation with the secondary antibody, the membrane was washed again for three times (5 min/time). Bound antibodies were visualized using 3,3′-diaminobenzidine (DAB) as a chromogenic substrate. [20]. The experimentation was performed on three independent biological replicates, i.e., from three individual buffaloes (n = 3) (S1 Fig).

### 2.3. Protein digestion

The protein samples used were extracted from tissues of flukes. Based on BCA assay quantification, total protein yield ranged from approximately 275–502 µg per sample. For digestion, **~** 150–165 µg of total protein per sample was reduced using 2 μL of 1 M dithiothreitol (20 mM) at 56 °C for 30 min. Then, alkylation was done by 6 μL of 500 mM iodoacetamide (final volume 100 mM) at room temperature for 30 min. Then, the samples were dissolved in a urea solution by adding 25 mM NH4HCO3. Finally, the digestion was conducted by Trypsin gold 1:100 enzyme-to-substrate ratio (Sigma-Aldrich, Hamburg, Germany) overnight at 37 °C.

### 2.4. LC-MS/MS analysis

Peptides were reconstituted in liquid chromatography mobile phase A and separated using a NanoElute ultra-high-performance liquid chromatography (UHPLC) system (Bruker Daltonics). Chromatographic separation was carried out on a 25-cm long reversed-phase nano-LC column (100 µm internal diameter) at a constant flow rate of 500 nL/min. Mobile phase A consisted of 0.1% formic acid and 2% acetonitrile in water, while mobile phase B contained 0.1% formic acid in acetonitrile. Peptides were eluted using a linear gradient of 6–24% B over 0–14 min, 24–35% B over 14–16 min, 35–80% B over 16–18 min, followed by a hold at 80% B for 18–20 min.

Following chromatographic separation, peptides were introduced into capillary electrospray ionization (ESI) source and analyzed using a timsTOF Pro time-of-flight (TOF) mass spectrometer. The ion source voltage was set to 1.75 kV. Data were acquired in data-independent acquisition parallel accumulation serial fragmentation (DIA-PASEF) mode. Full MS scans were collected over an m/z range of 300–1500, followed by 20 PASEF MS/MS scans per cycle. Fragment ion spectra were acquired in the m/z range of 400–850 using isolation windows of 7 m/z. Before LC–MS/MS analysis, peptide samples undergo normal cleanup methods to exclude low-molecular-weight impurities and salts.

### 2.5. Database search

DIA datasets were processed using DIA-neural networks (DIA-NN) (v1.8.1) under default parameters (https:github.com/vdemichev/DiaNN/releases/tag/1.8.1). DIA-NN’s built-in normalization algorithm was used to normalize the data in order to account for systematic technical variation between runs. Tandem mass spectra were searched against the Uniprot database (https;//www.uniprot.org/; *Fasciola gigantica;* Taxon ID: 46835; 13,102 entries) Trypsin/P was specified as the proteolytic enzyme allowing upto one missed cleavage. Fixed modifications encompassed N-terminal methionine excision and cysteine carbamidomethylation. A theoretical spectral library was computationally generated via a deep learning algorithm, and a decoy reverse library was incorporated to quantify the false discovery rate (FDR) arising from random matches. The false discovery rate (FDR) was strictly controlled at ≤1% at both peptide and protein levels. To reduce false positive identifications, common laboratory contaminants (including keratins and trypsin-derived peptides) were filtered during data processing. For downstream analysis, only high-confidence protein identifications passing DIA-NN scoring thresholds were retained. Missing values were handled using DIA-NN default settings, where protein inference was based on consistent peptide detection across runs, minimizing the impact of stochastic missingness inherent to DIA datasets. Standard bioinformatic and statistical pipelines incorporated into DIA-NN and downstream analysis tools were used for statistical analyses, with relevant significance criteria applied where applicable.

### 2.6. Bioinformatics analyses

The detailed protein profile of *F. gigantica* was subject to multiple computational analyses. Involvement of the proteins in distinct biological pathways was systematically evaluated. GO annotation was performed using the g: Profiler web server (https://biit.cs.ut.ee/gprofiler/) for functional enrichment. Graphical representations were generated using GraphPad Prism software. All matched and assigned protein sequences in this study were initially analyzed using SignalP 5.0b [39], to detect classical signal peptides. Sequences lacking such peptides were subsequently examined with TMHMM 2.0c [40] to predict potential transmembrane regions. Subcellular localization across multiple cellular compartments was then inferred using DeepLoc 2.0 [41], which integrates sequence-derived features. To contextualize protein associations, the STRING database (https://string-db.org/), an integrative platform cataloguing both functional and physical protein‒protein interactions, was used for enrichment analyses and network modeling. The molecular mechanisms behind proteins can be easily interpreted by using route analysis to condense a large list of proteins onto a small list of pathway knowledge maps [42].

## 3. Results

**Fig 1:**
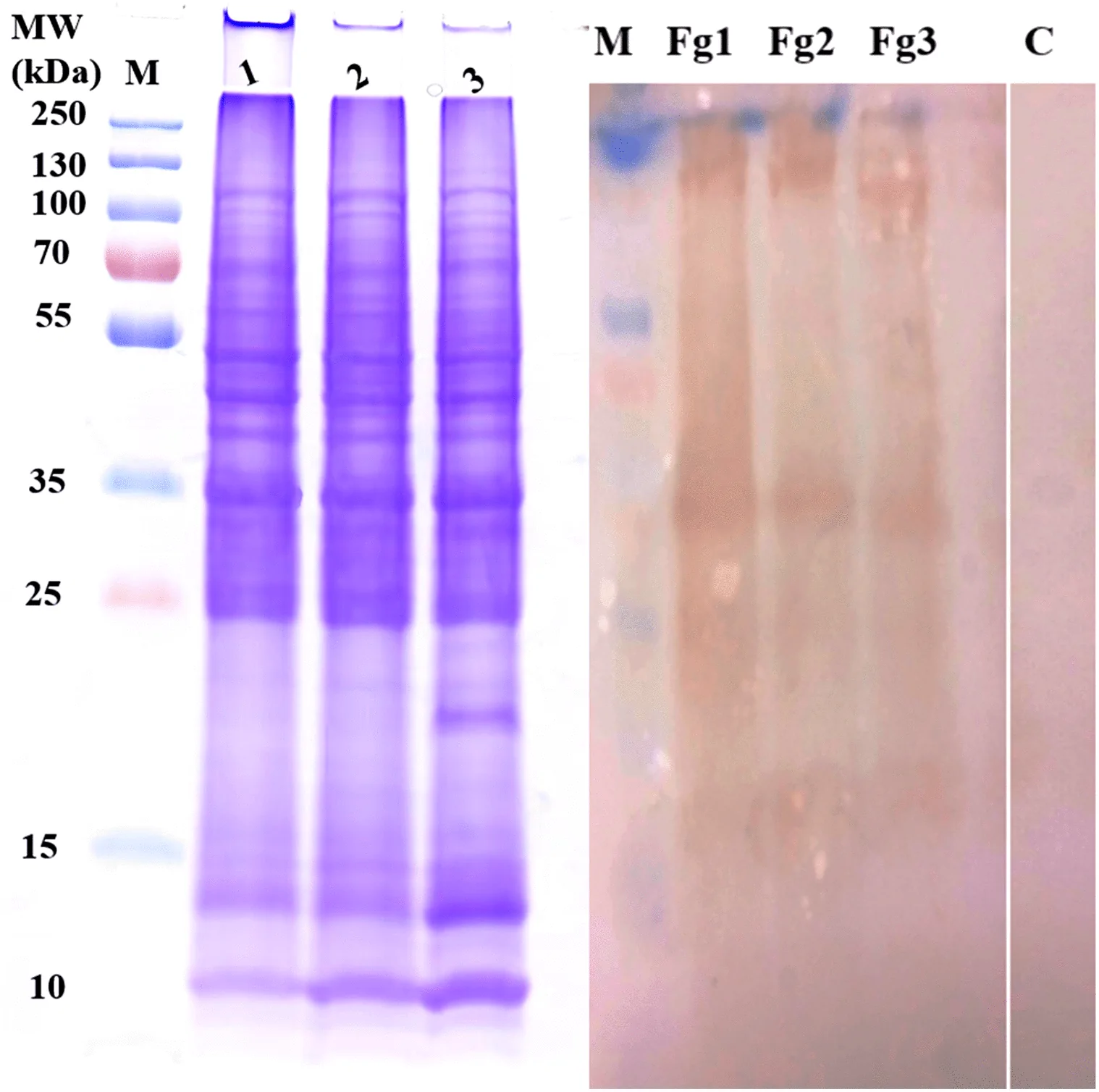
Sodium dodecyl sulfate–polyacrylamide gel electrophoresis (SDS–PAGE) and western-blot analysis of three adult *Fasciola gigantica.* M: molecular weight protein marker (kDa). 1-3: protein contents from three adult flukes (n = 3). Fg1-Fg3: immunogenicity of proteins from three adult flukes with specific antibodies.

As shown by SDS-PAGE analysis of *F. gigantica* samples (Fg1–Fg3), reproducible and well-resolved protein profiles with a range of approximately 10–250 kilo-dalton (kDa) were revealed, suggesting an effective and reliable protein extraction. Western blot analysis of all *F. gigantica* samples showed smear-like immunoreactivity across a wide molecular weight range, indicating that the antibodies recognized various antigenic components within protein extracts, and providing complementary evidence consistent with the intricate protein profile observed in the proteomic analysis (Fig 1).

The samples and control were resolved on the same SDS gel and transferred under identical conditions. After transfer, the membrane was sectioned, and the samples and negative control were probed separately with antibodies to ensure clear signal interpretation. This separation was performed exclusively at the post-transfer stage and does not affect data normalization. As the Protein signal intensities were adjusted or normalized to account for changes in sample loading and detection performance, ensuring reliable comparison across samples, then such diffusive patterns frequently linked to glycoproteins, proteases, and other ESPs in helminth proteomes, reflecting the diversity of immunogenic targets. The immunoreactivity was specific to *F. gigantica* proteins, and no bands were observed in control lane indicating the absence of nonspecific binding.

**Fig 2:**
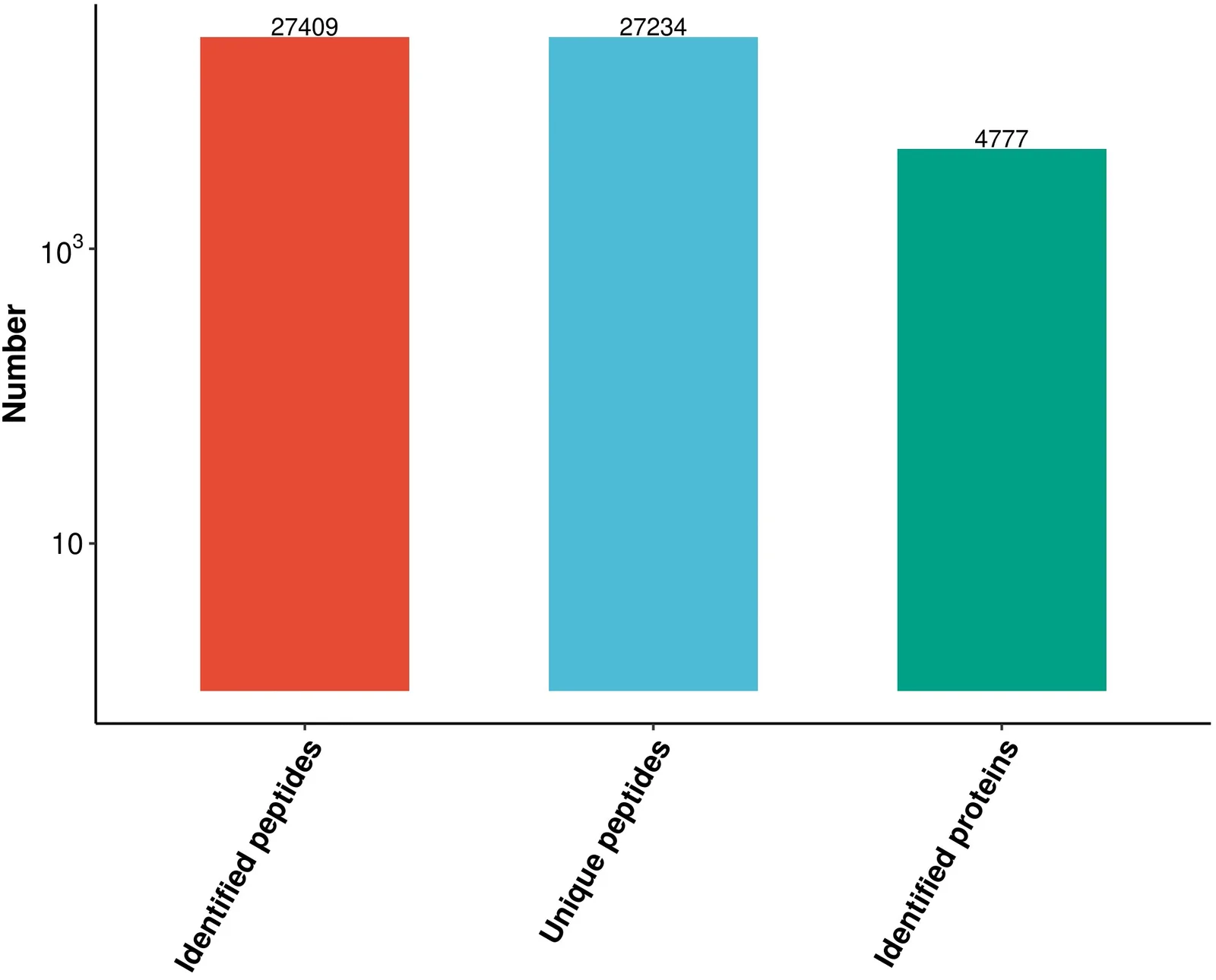
Liquid chromatography–tandem mass spectrometry (LC–MS/MS) –based identification of peptides and proteins of adult flukes. Identified peptides (red bar) are all the peptide-spectrum matches, while unique peptides (blue bar) are the non-redundant peptide sequences that help identify proteins with confidence. The third bar (green) indicates the total number of matched proteins based on peptide evidence.

Total 4,777 different proteins were matched by the spectrum database search, which yielded 27,409 peptides in total, including 27,234 unique sequences (Fig 2). No comparable proteins were found. So the proteins found were exclusive to the dataset under analysis (Table 1) (S2 Table).

**Table 1:** Summary of spectrum database search analysis.

Identified peptides	Unique peptides	Identified proteins	Comparable proteins
27,409	27,234	4,777	0

Note: The table summarizes the output of database search performed on Liquid chromatography–tandem mass spectrometry (LC-MS/MS) based spectra.

Out of these 271 potential proteins were predicted to be as ESPs.

**Fig 3:**
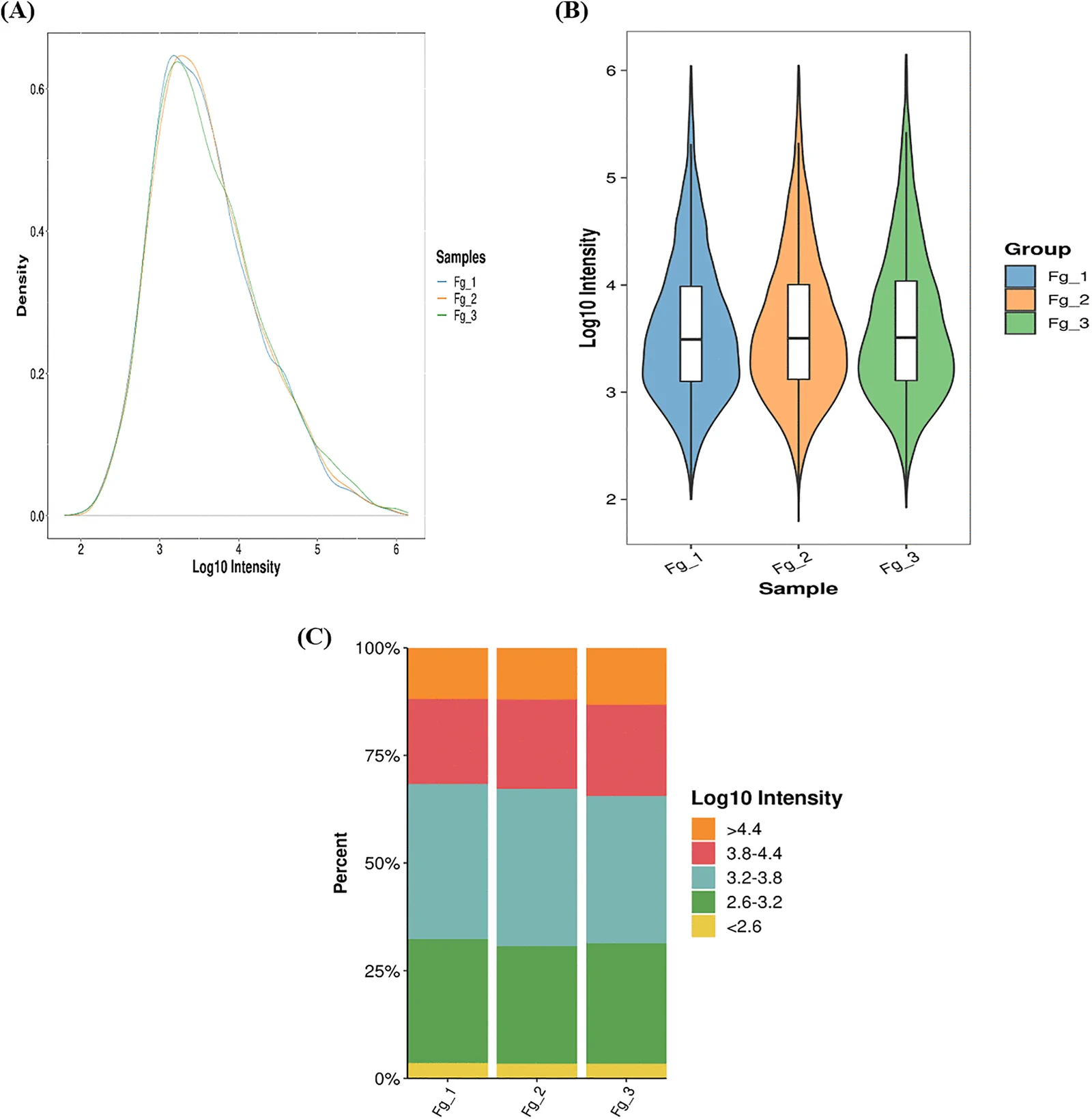
Protein intensity distribution and variability. **(A)** Intensity density distribution, three curves represent the frequency of assigned proteins. **(B)** Violin plot, wider sections shows high protein abundance. **(C)** Stack bar plot, x-axis represents samples, and y-axis illustrates the percent of proteins.

The distribution of protein intensities of the three biological replicates was analyzed. Density plots were generated using log10-transformed protein intensity measures to depict the distribution of protein abundances across biological replicates. The density plots (Fig 3, panel A) revealed overlapping unimodal curves, indicating high technical precision and reproducibility. Violin plots (Fig 3, panel B) demonstrated consistent protein abundance across replicates, with comparable medians, interquartile ranges, and overall distribution patterns, while outliers reflected proteins with markedly higher abundance. The stacked bar plots (Fig 3, panel C) further illustrated uniform protein proportions across defined intensity bins. The distribution of proteins at various defined intensities, showed that the majority of proteins were found in the moderate intensity range, with comparatively fewer proteins falling into the extreme low or high abundance categories (Fig 3).

**Fig 4:**
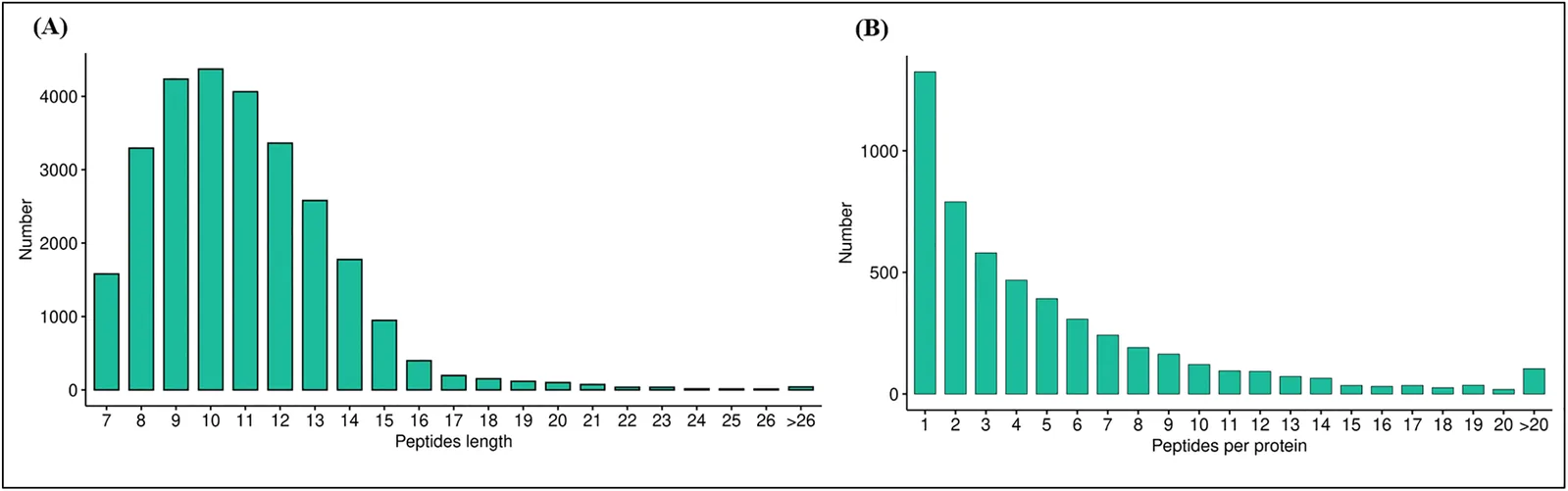
Distribution of peptide lengths and peptides per protein. **(A)** The number of peptides for each protein are shown in a bar graph. Only a small percentage of proteins are supported by numerous peptide hits, whereas the majority are supported by one or two peptides. This distribution shows the protein abundance and detection sensitivity of the examined samples. **(B)** Peptide lengths found in the MS study are displayed as a graph with a number distribution. For the best ionization and fragmentation efficiency during MS/MS analysis, this length range is used.

Reliable and substantial proteome coverage was supported by the peptide length distribution, which revealed that the majority of detected peptides were within the optimal range and that most proteins were supported by several peptides. Collectively, these analyses confirmed consistent signal strength, low technical variance, and the robustness of the proteomic dataset for downstream bioinformatics investigations (Fig 4).

**Fig 5:**
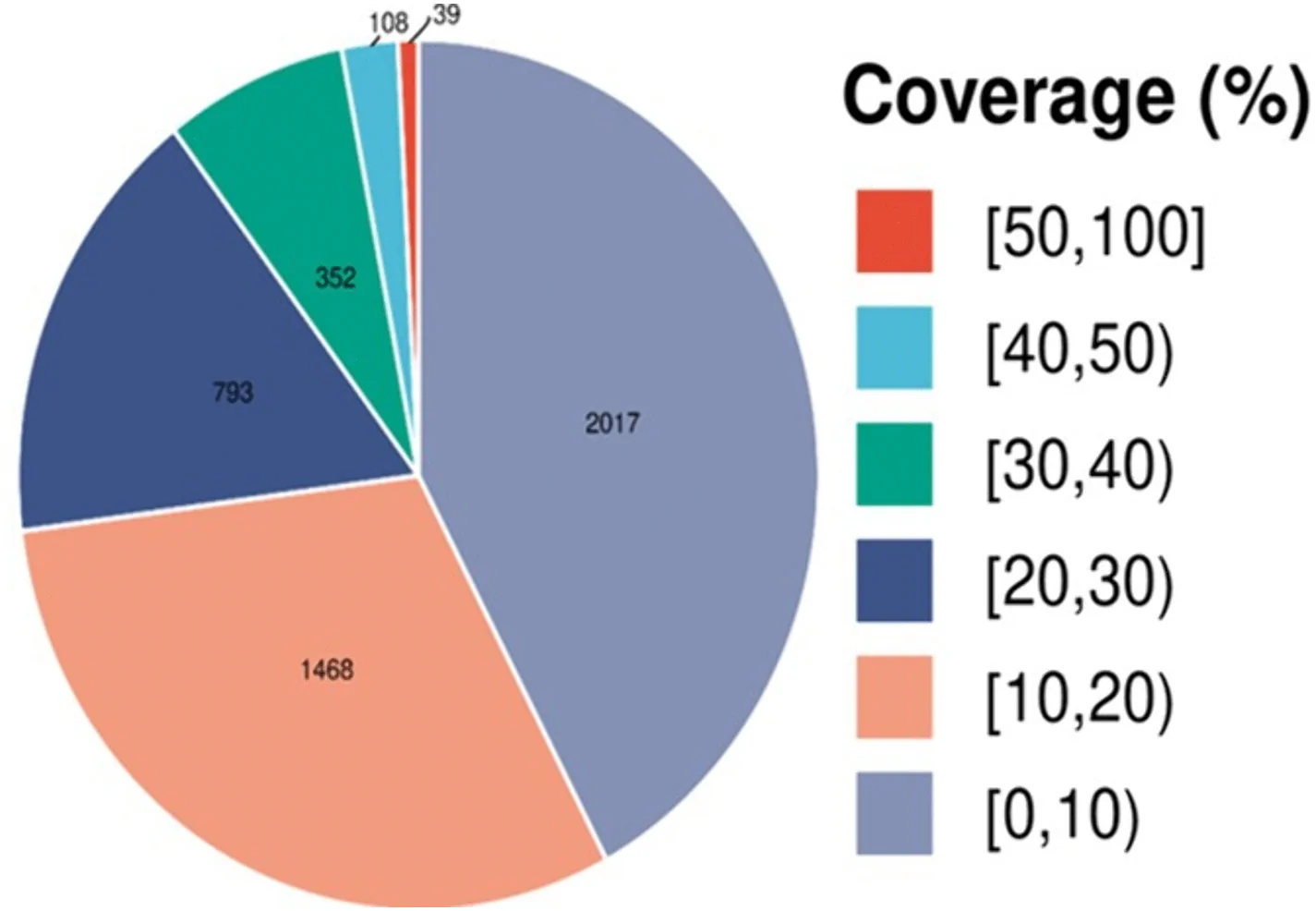
Distribution of protein sequence coverage (%) determined through LC-MS/MS analysis. The display of the distribution of proteins represents every color segment corresponds to a certain range of amino acid’s sequence coverage by peptides.

Majority of the proteins analysis showed partial sequence coverage, mostly between 0 and 10%, which is consistent with normal large-scale proteomics datasets. A smaller proportion of proteins has moderate (20–40%) or high (>40%) coverage, whereas fewer proteins have coverage between 10% and 20%. The percentage of each protein’s amino acid sequence that is matched by peptides found by LC-MS/MS analysis is known as sequence coverage. The distribution showed that peptide representation differs throughout the proteome, reflecting variations in the detectability and quantity of proteins. (Fig 5).

### 3.1. Excretory–secretory proteins prediction

Out of all the 4,777 proteins in *F. gigantica* species, 271 have been predicted as Excretory Secretory proteins (S3 Table) (S4 Table).Then these proteins were further analyzed using different software to determine their biological, molecular, and cellular functions. This combined experimental and computational approach provides the first insight into the somatic proteome and predicted secretome of *F. gigantica* in this region. Molecular functions of the predicted ESPs are presented in Table 2, while biological processes and cellular components are summarized in Tables 3 and 4, respectively.

**Table 2:** Molecular functions of predicted excretory–secretory proteins (ESPs).

Functional Class	Representative GO Terms	GO Term Name	No. of proteins
**Protease activity**	GO:0004175	endopeptidase activity	12
	GO:0008233	peptidase activity	15
	GO:0008234	cysteine-type peptidase activity	8
	GO:0008236	metallopeptidase activity	5
	GO:0004252	serine-type endopeptidase activity	6
	GO:0004197	cysteine-type endopeptidase activity	4
**Hydrolase activity**	GO:0016787	hydrolase activity	10
	GO:0016798	hydrolase activity, acting on glycosyl bonds	9
**Protease inhibitor activity**	GO:0004866	endopeptidase inhibitor activity	7
	GO:0004857	enzyme inhibitor activity	3
	GO:0061135	endopeptidase inhibitor activity	3
	GO:0061134	peptidase inhibitor activity	2
**Structural/ ECM**	GO:0005201	structural molecule activity	8
**Molecular adaptor/ regulator**	GO:0017171	guanyl-nucleotide exchange factor activity	3
**Carbohydrate processing**	GO:0004553	alpha-glucosidase activity	5

Note: Predicted ESPs were categorized using Gene Ontology-based molecular function analysis.

**Table 3:** Biological Processes of Predicted ESPs.

Functional Class	Representative GO Terms	GO Term Name	No. of proteins
**Regulation of biological processes**	GO:0065009	biological regulation	20
**Proteolysis & protein catabolism**	GO:0006508	Proteolysis	18
**Vesicle-mediated transport**	GO:0032411	protein transport	12
**Response to stimulus/ stress**	GO:0051604	protein stabilization	3
**Signal transduction**	GO:0007267	cell-cell signaling	9
**Protein folding/ quality control**	GO:0006457	protein folding	6
**Ion transport**	GO:0022898	Ion-transmembrane transport	5
**Lipid metabolism**	GO:0006629	lipid metabolic process	4

Note: Predicted ESPs were categorized based on the biological pathways in which they are involved using GO based biological process analysis. Parasite survival, host contact, and metabolism are all related to these processes.

**Table 4:** Cellular Components of Predicted ESPs.

Cellular Localization	Representative GO Terms	GO Term Name	No. of proteins
**Membrane & membrane-associated**	GO:0016020	Membrane	25
**Extracellular region**	GO:0005576	extracellular region	18
**Intracellular organelles**	GO:0043235	Organelle	12
**Endoplasmic reticulum**	GO:0005783	ER membrane	10

Note: This categorization is based on the cellular divisions associated with ESPs secretion and functions.

### 3.2. Bioinformatics analyses

#### 3.2.1. Gene ontology (GO) analysis.

**Fig 6:**
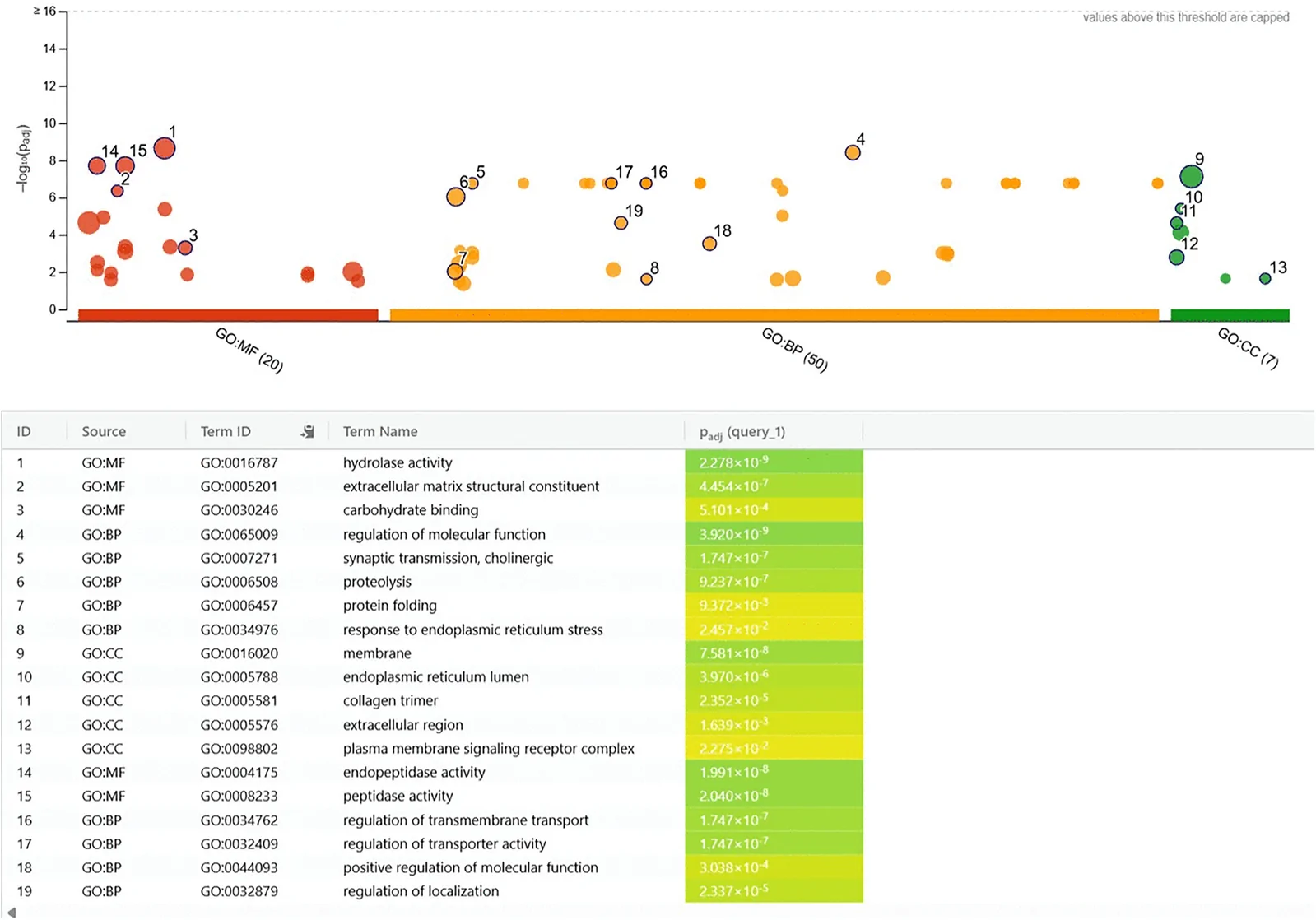
Gene Ontology (GO) annotation of excretory–secretory proteomic data (ESPs). The proteomic profile was grouped into biological processes, cellular components, and enriched molecular activities. According to their GO keywords, three groups were depicted as: cellular component (green), biological process (orange), and molecular function (red). Each dot stands for a GO word, and the dot’s size indicates the importance of the protein enrichment. The top 15 GO terms are mentioned in the table above.

Significant associations between the discovered proteins and a variety of molecular activities, biological processes, and cellular components were observed by GO enrichment analysis. GO enrichment analysis was carried out with common functional annotation tools. Adjusted p-values (multiple testing correction applied by g:Profiler) were used to assess the statistical significance of GO terms, and only significantly enriched GO categories were selected for downstream analysis and visualization. The results are presented separately for somatic proteins and putative excretory–secretory proteins (Fig 6).

Following the GO analysis of ESPs, a subsequent Gene Ontology (GO) enrichment analysis of the somatic (whole) proteome was performed to further elucidate the functional landscape of non-secretory proteins. Significant representation across molecular function, biological process, and cellular component categories was revealed by GO enrichment analysis of the somatic proteome (S5 Fig). Catalytic activity, small molecule binding, and protein binding were the most enriched terms in molecular functions, suggesting that proteins involved in interactions and enzymatic processes predominate.

Cellular localization, small molecule metabolic processes, cellular component organization or biogenesis, mRNA metabolic activities, and protein maturation were all enriched in biological process analysis. According to their intracellular location and participation in structural assemblies, proteins were primarily linked to the cytoplasm in the cellular component category, followed by supramolecular complexes and motile cilia. (S5 Fig).

#### 3.2.2. Enrichment analysis.

**Fig 7:**
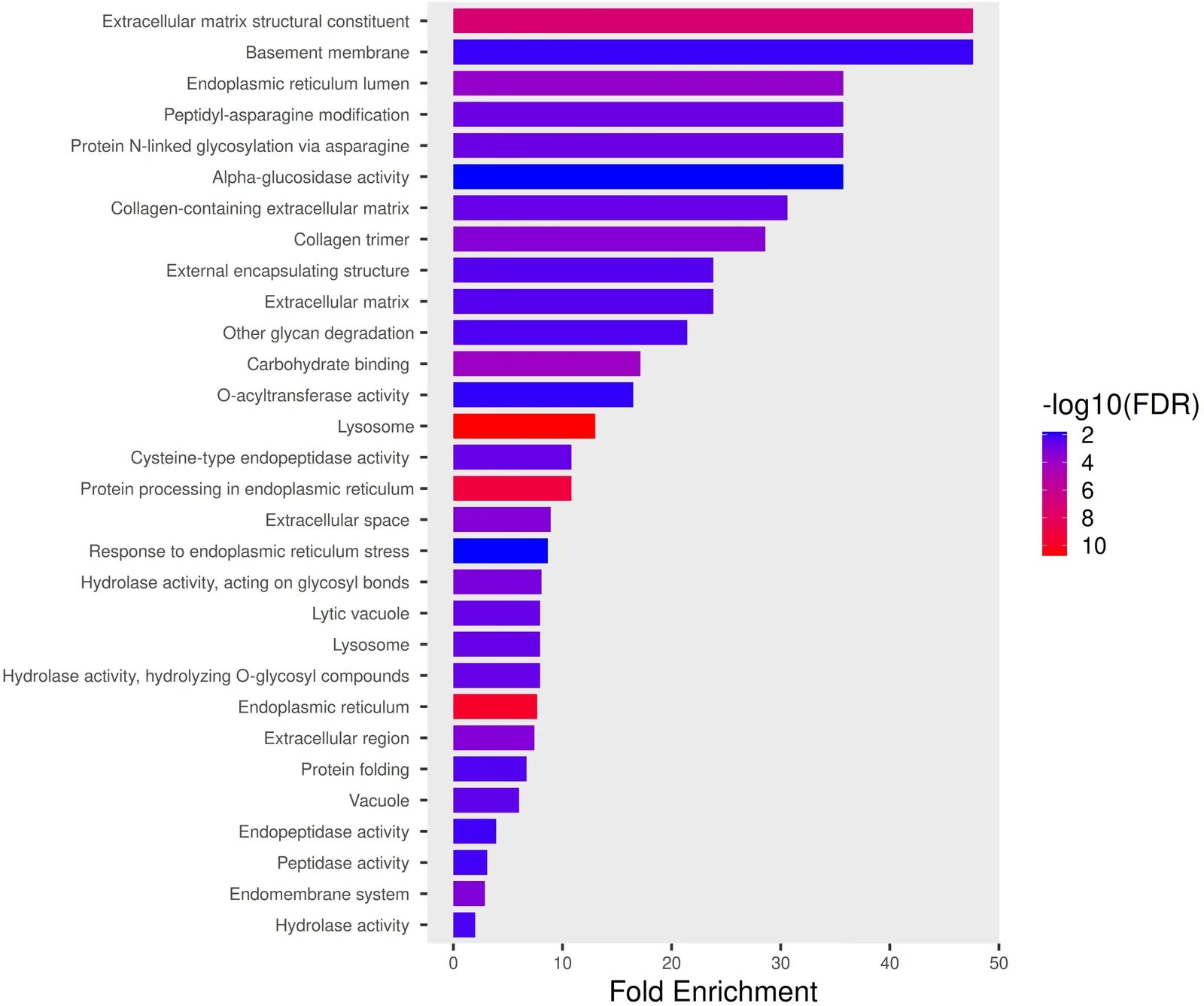
Fold enrichment analysis of proteomic profiles and their functional pathways. The X-axis represents fold enrichment, and the Y-axis represents highly enriched pathways. Color intensity shows the statistical significance. Highly enriched pathways are towards the right and darker.

This enrichment study focused on mechanisms that promote host tissue invasion, immunological regulation, nutrition acquisition, and survival within the host environment, reflecting the highly specialized molecular machinery of the parasite that supports *F. gigantica* lifecycle. Enrichment in amino acid transport and metabolism, glycolysis/gluconeogenesis, lipid metabolism, and proteolysis pathways facilitate efficient intake of host-derived nutrients. These enhanced pathways may help find possible treatment targets or diagnostic indicators for the management of human and animal infections caused by *F. gigantica* (Fig 7).

#### 3.2.3. STRING analysis.

**Fig 8:**
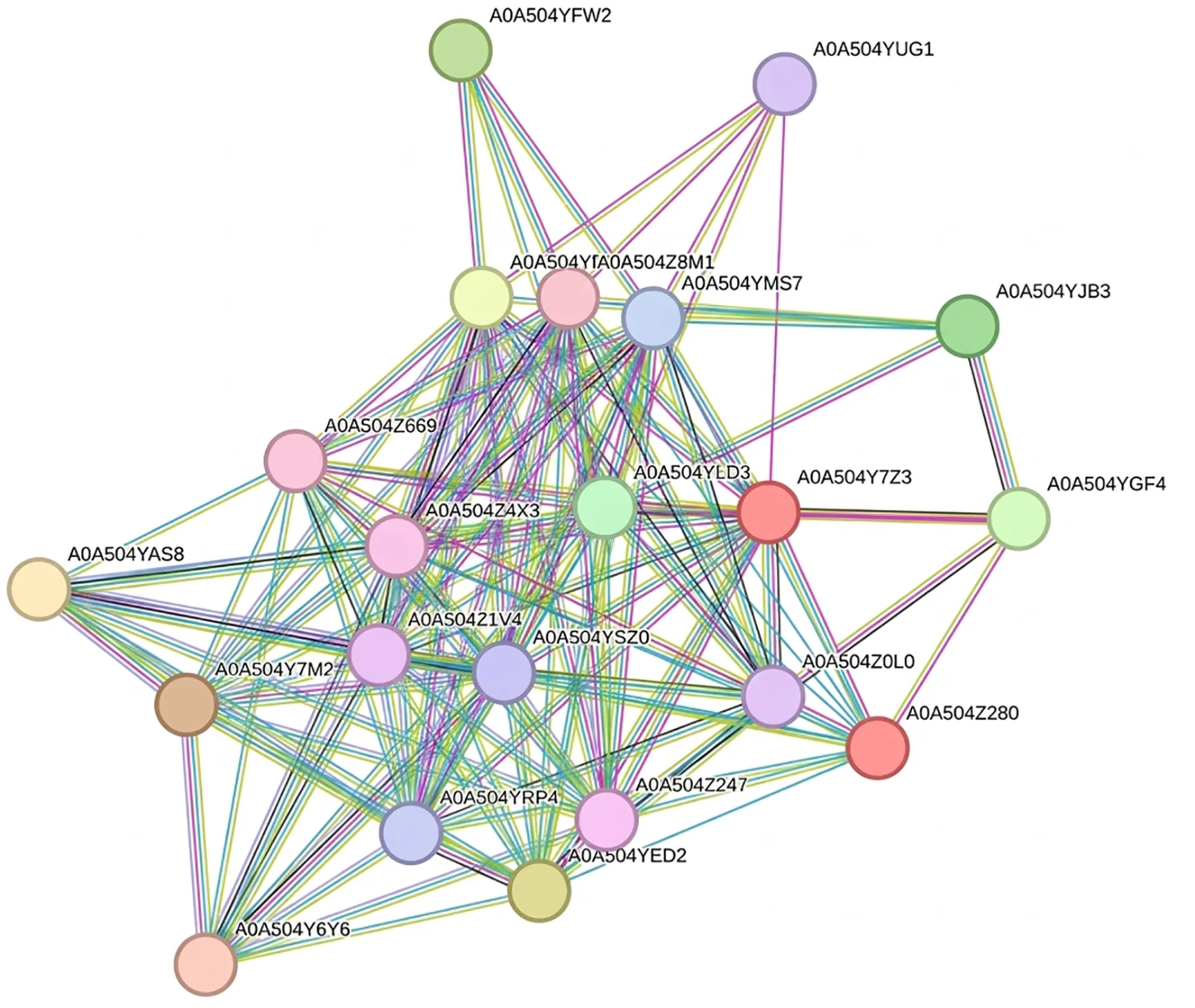
Protein-Protein interactions (PPI) via STRING analysis. Each node represents an identified protein and edges show functional associations. Stronger edges indicate a high confidence score.

The protein interaction landscape of *F. gigantica* is visually represented by this string analysis, which may prove useful for proteomics studies, medication development, and comprehending the biology of the parasite (Fig 8). The STRING database for *F. gigantica* was used for protein–protein interaction analysis, which included both known and predicted interactions with a medium confidence level (0.4). With major hub proteins interacting with several partners, the network demonstrated a high degree of connectedness overall, suggesting functional interdependence and offering more insight into the structure and biological significance of the proteome. Motor proteins like dynein and kinesin were observed to be important parts of a large cluster of cytoskeleton-related proteins. These proteins are known to be crucial for cytoskeletal dynamics and intracellular transport, and their robust connections imply coordinated participation in cellular functions and structural organization.

The existence of connections between dynein and kinesin emphasized active transport processes and supports the cytoskeletal network’s functional significance in the system under study. Such cytoskeletal and motor protein dynamics may support intracellular survival, parasite movement, and host cell architecture regulation in the context of host–parasite interactions. Additionally, these connections may facilitate the movement of chemicals derived from parasites, allowing for efficient host manipulation and environmental adaption. Several kinesin family members, such as kinesin light chain and kinesin family member 5, generated the majority of the network, indicating a crucial function for microtubule-based transport pathways. Other important interacting proteins were found to be involved in intracellular positioning and cytoskeletal architecture, including Nuclear Distribution protein NudE 1 and Fasciculation and elongation protein zeta. The control of cytoskeletal dynamics through phosphorylation-dependent signaling pathways was further suggested by regulatory proteins such as casein kinase and c-Jun N-terminal kinase-interacting protein 4.

Host–parasitic cytoskeletal interactions are particularly relevant, as numerous parasite cytoskeletal proteins are antigenic and can activate a host immune response upon release through tissue injury or excretory/secretory products. These proteins represent potential but neglected prospects for the creation of novel vaccination targets and diagnostic biomarkers [43].

### 3.3. Statistical analysis

The majority of GO terms exhibited robust enrichment, with adjusted *p*-values < 0.01. The two factors, that are most important for metabolic adaptability and parasite survival, are hydrolase activity and molecular function regulation.

## 4. Discussion

The somatic proteome and predicted ESPs of *F. gigantica* in this study exhibited substantial functional diversity, suggesting potential roles in host-parasite interactions and parasite adaptation within host environment.

To capture both classical and non-classical secretory proteins, proteins were classified as putative ESPs if they were predicted to be extracellular by DeepLoc, possessed or lacked an N-terminal signal peptide (SignalP), and were devoid of transmembrane helices as predicted by TMHMM. Classically secreted ESPs were defined as proteins translocated into the endoplasmic reticulum via a single N-terminal signal peptide, typically 18–25 amino acids in length.

Total 4,777 proteins including 271 excretory/secretory components provided the most thorough characterization of the *F. gigantica* proteomic profile from geographical region of Pakistan. While *F. gigantica* is considered to be the prevalent species in Punjab, genetic identification of the studied specimens was not performed. All flukes were recovered from Nili Ravi breed of buffaloes grown locally in municipal and surrounding slaughterhouse sites, providing context for potential species attribution and environmental exposure. The proteomic dataset comprised approximately ten times more proteins than those previously reported in *Fasciola* spp. [31]. Somatic protein extracts were prepared for SDS–PAGE, Western blotting, and LC–MS/MS analyses. Protein profiling was made possible by the combination of high-resolution LC-MS/MS, multi-database analysis, and optimal sample preparation.

A wide variety of ES proteins have been predicted, which adds to our knowledge of the molecular mechanisms that *F. gigantica* uses during infection and validates earlier discoveries in trematode biology. We also performed prediction analyses to predict putative ESPs and subcellular localization of proteins by using SignalP 5.0b. The discovery of large dataset of classical ES proteins in this study, including proteinases (cathepsin B-like cysteine proteinases), calreticulin, C-type lectins, thioredoxin domain-containing proteins, and protein disulfide isomerases, is noteworthy. Cathepsin L proteases have been identified as promising vaccine candidates for *F. hepatica* [44]. Cathepsin L proteases are well-known virulence factors in *Fasciola* spp., where they facilitate hemoglobin breakdown and aid parasite adaptability to the host environment [45]. These proteases play crucial roles in the interactions between the host and the parasite after being secreted into the host tissues. In addition to proteases, the parasites secrete a variety of proteins with antioxidant properties that are also tightly controlled based on how they move through host tissues: glutathione S-transferases (GSTs), redox enzymes (peroxiredoxin, thioredoxin, and protein-disulfide isomerase), and fatty acid-binding proteins (FaBPs 1,-3 and Fh15). Fluke immune avoidance mechanisms have been linked to these substances [46,47].

Specifically, cathepsins, associated peptidases and C-type lectins, identified in this study, are known virulence factors that may contribute to immune evasion and host tissue destruction by degrading collagen and fibronectin, highlighting their potential as targets for vaccines and medications [48]. C-type lectins play a variety of functional roles in cell death, immunology, and physiology. They are extensively dispersed and frequently expressed in many helminths [16,49]. In line with previous reports on trematode proteomic study, ES molecules were essential for tissue invasion, nutrient acquisition, and host immune response modulation [50].

Notably, a number of proteins with adhesion and signaling properties are also observed, such as ECM, thrombospondin-2, proteins with laminin N-terminal domains, and different proteins with immunoglobulin-like and EGF-like domains. By modifying biological process and aiding parasite adhesion in host tissues, these proteins may promote persistent infection. The parasite’s strategy to neutralize host-derived reactive oxygen species and ensure life in the hostile host environment is highlighted by the antioxidant enzymes like glutathione-related proteins, thioredoxin peroxidases, and peroxiredoxins. Reactive oxygen species (ROS) cause lipid peroxidation, tegumental damage, and parasite death. ROS comprises superoxide, hydrogen peroxide (H_2_O_2_), and the hydroxyl radicals. Superoxide dismutase (SOD) converts to H_2_O_2_, and subsequently catalase and glutathione peroxidase (GPx) detoxify H_2_O_2_, by oxidation and coupling with glutathione, respectively. These three main antioxidant enzymes work in succession to reduce free radicals. Because GPx level is low and catalase is absent, peroxiredoxins (Prx) serve as the primary detoxifying enzyme in helminth parasites, such as *Fasciola* spp. [51].

The somatic proteome’s GO enrichment profile emphasizes a significant majority of catalytic and binding functions, highlighting the organism’s metabolically active and functionally diverse characteristics. A tightly controlled network supporting cellular homeostasis, biosynthesis, and post-transcriptional regulation is suggested by the notable levels of pathways linked to small molecule metabolism, mRNA processing, and protein maturation. Significantly, an active intracellular transport system and cytoskeletal organization—both necessary for preserving cellullar structures and dynamic protein distribution—are indicated by the predominance of microtubule-based processes and cellular localization pathways (S5 Fig). The finding of proteins in this study is associated with the extracellular matrix and structural constituents, such as collagens, heat-shock (HSP70), cadherin-like proteins, cytoskeletal and vitelline protein B2 (not ESPs). Among the antigens found in *E. multilocularis* protoscoleces, cytoskeletal and heat-shock proteins (HSPs) were shown to be immunodominant [52]. Collagens are proteins with immunogenic and antigenic characteristics [53,54].

These constituents may play a role in the growth and integrity of the parasite’s tegument. These two processes are essential for the parasite’s survival in the host. In addition, the parasite’s dependence on protein processing and secretion machinery to sustain functional secretory pathways is demonstrated by the presence of lectins, glycoprotein-modifying enzymes, and Endoplasmic reticulum (ER) chaperones.

The top proteins in PPI interactions are kinesin mediated proteins, also identified in this study. To survive in the challenging host environment, parasites must have both structural integrity and motility, which are supported by the movement of cytoskeletal components mediated by kinesin [55]. Proteomic analyses of predicted ESPs and somatic fractions have identified kinesins, indicating their involvement in both intracellular transport and secretion-related processes, despite the paucity of studies particularly on *F. gigantica.*

In contrast to the ESP dataset, which is typically enriched in extracellular and host-interaction-related functions, the somatic proteome reflects fundamental intracellular processes required for parasite survival and maintenance. This distinction highlights the functional compartmentalization between secretory and non-secretory proteomes, providing complementary insights into both host–parasite interactions and intrinsic cellular biology. We also identified a subset of proteins in this study, such as annexins, a tetraspanins, carbonic anhydrase, and a CD59-like orthologue, that are also shared with the *schistosoma* and *F hepatica* tegument. These proteins probably play a role in the host-parasitic interface, especially in immune regulation and complement-mediated damage inhibition, which let the parasite survive inside the host.

This proteomic characterization of putative ES and somatic proteins deepens our comprehension of the molecular processes that underlie the pathophysiology of *F. gigantica*. The wide range of functions highlights how proteolytic breakdown, immunological regulation, antioxidant protection, and structural adaptability all work together to help parasites survive. To assess the functions of these proteins and evaluate their potential as candidates for diagnostics, vaccines, or innovative therapeutic approaches, future research combining functional assays, immunological characterization, and host–parasite interaction models is crucial.

The multifunctional nature of predicted *F. gigantica*’s ES proteins is highlighted by the combined proteomic and functional enrichment analyses, which connect pathogenesis to host tissue contact, metabolic control, immunological evasion, and enzymatic destruction.

These findings provide significant biological insights; nevertheless, specific limitations must be acknowledged in the interpretation of the data. Although, the somatic proteins of *F. gigantica* were the primary focus of this study, the results provide a solid basis for further investigation of full range of proteoforms, which may further clarify host-parasite interactions. One possible limitation of this study is that putative ESPs were inferred from homogenized flukes using bioinformatic prediction and functional annotation, which might not accurately reflect the entire range of proteins being released into the host biliary environment.. However, somatic proteins were also examined and discussed to offer a more thorough functional viewpoint and an integrated understanding of parasite biology.

This comprehensive study is also limited by the inherent constraints of the mass spectrometry platform. The bioinformatics tools offer valuable insights into potential immunomodulatory roles, therefore targeted experimental validation is required to further validate biological functions. Furthermore, molecular confirmation of *F. gigantica* and differentially abundant proteins across various developmental stages were not performed because this work concentrated on somatic proteins and in silico predicted ESPs from a single infection stage. A limitation of this study concerns the limited number of biological replicates, indicating that the data should be regarded as exploratory rather than entirely quantitative. One more limitation concerns that like other peptide-based (bottom-up) LC–MS proteomic methods, the ability to identify proteins may be affected by the efficiency of ionization and the dynamic range, which could make it easier to find more abundant peptides.

## 5. Conclusions

In this study, 4,777 proteins were inferred from the proteomic profile of adult *F. gigantica* flukes after SDS-PAGE, western blotting, and mass spectrometry analysis (Table 1). Out of which 271 proteins were predicted to be ES in nature. The results were passed through different bioinformatics analyses, which imply that predicted ES proteins have a variety of functions in molecular and biological pathways, i.e., in both host interaction and parasite survival. The present study not only characterized the proteomic profile but also highlighted the functional significance of somatic and ESP proteins in host–parasite interactions. ECM and basement membrane-related proteins are present, which suggests that they are involved in tissue adhesion, penetration, and host cell architectural modification. Protein stability and immune evasion depend on an active secretory machinery with significant post-translational processing, which is reflected in proteins confined to the ER lumen and those undergoing peptidyl-asparagine modification.

## Supporting information

S1 Fig Raw images of gel and western blot. (TIF)

S2 Table Detailed list of identified proteins. (XLSX)

S3 Table Predcited signal peptide cleavage positions. (XLSX)

S4 Table *In-silico* prediction of all putative ES_proteins. (XLSX)

S5 Fig Gene Ontology (GO) annotation of whole proteome. (TIF)

## Abbreviations

*F. gigantica*: *Fasciola gigantica*
*F. hepatica*: *Fasciola hepatica*
ESPs: Excretory-Secretory proteins
SDS-PAGE: Sodium dodecyl sulfate–polyacrylamide gel electrophoresis
LC-MS/MS: Liquid chromatography–tandem mass spectrometry
kDa: Kilo dalton
GO: Gene Ontology
